# Comparison of an Innovative Rehabilitation, Combining Reduced Conventional Rehabilitation with Balneotherapy, and a Conventional Rehabilitation after Anterior Cruciate Ligament Reconstruction in Athletes

**DOI:** 10.3389/fsurg.2017.00061

**Published:** 2017-11-07

**Authors:** Laetitia Peultier-Celli, Didier Mainard, Frank Wein, Nicolas Paris, Patrick Boisseau, Alexandre Ferry, René Gueguen, Isabelle Chary-Valckenaere, Jean Paysant, Philippe Perrin

**Affiliations:** ^1^Faculty of Medicine and UFR STAPS, University of Lorraine, EA 3450, Development, Adaptation and Handicap, Villers-lès-Nancy, France; ^2^Laboratory for the Analysis of Posture, Equilibrium and Motor Function (LAPEM), University Hospital of Nancy, Vandoeuvre-lès-Nancy, France; ^3^Department of Orthopedics and Trauma Surgery, University Hospital of Nancy, Nancy, France; ^4^Department of Orthopedics Surgery, Médipôle Gentilly-Saint-Jacques, Maxéville, France; ^5^Institut de Formation en Masso-Kinésithérapie, Nancy, France; ^6^Department of Rheumatology, University Hospital of Nancy, Vandoeuvre-lès-Nancy, France; ^7^IMoPA, UMR 7365 CNRS, Vandoeuvre-lès-Nancy, France; ^8^Regional Institute of Physical Medicine and Rehabilitation, Nancy Cedex, France

**Keywords:** anterior cruciate ligament injury, rehabilitation, balneotherapy, postural control, proprioception, sports injury

## Abstract

**Background:**

Instability of the knee, related to anterior cruciate ligament injury, is treated by surgical reconstruction. During recovery, a loss of proprioceptive input can have a significant impact. Few studies have evaluated the benefits of rehabilitation of the knee in aquatic environment on functional outcomes.

**Objective:**

This study aimed to compare an innovative rehabilitation protocol combining reduced conventional rehabilitation with aquatic rehabilitation, with a conventional rehabilitation, according to the National French Health Authority, in terms of kinetics, development of proprioceptive skills, and functional improvement of the knee.

**Methods:**

67 patients, who were amateur or professional athletes, were randomized into two groups: 35 patients followed the conventional rehabilitation protocol (Gr1) and 32 patients followed the innovative rehabilitation protocol (Gr2). Patients were evaluated before surgery, and at 2 weeks, 1, 2, and 6 months after surgery using posturography, and evaluation of muscular strength, walking performance and proprioception. This study is multicenter, prospective, randomized, and controlled with a group of patients following conventional rehabilitation (level of evidence I).

**Results:**

For the same quality of postural control, Gr2 relied more on somesthesia than Gr1 at 6 months. The affected side had an impact on postural control and in particular on the preoperative lateralization, at 2 weeks and at 1 month. Lateralization depended on the affected knee, with less important lateralization in Gr2 preoperatively and at 1 month. The quadriceps muscular strength was higher in Gr2 than in Gr1 at 2 and 6 months and muscle strength of the external hamstring was greater in Gr2 than in Gr1 at 6 months. The isokinetic test showed a greater quadriceps muscular strength in Gr2. Gr2 showed a greater walking distance than Gr1 at one month. Gr2 showed an improvement in the proprioceptive capacities of the operated limb in flexion for the first 2 months.

**Conclusion:**

The effectiveness of the innovative rehabilitation program permits faster recovery, allowing for an earlier return to social, sporting, and professional activities. Faster retrieval of knee function following aquatic rehabilitation would prevent both short-term risk of lesions of the contralateral limb due to overcompensation and long-term risk of surgery due to osteoarthritis.

**Registration of clinical trials:**

NCT02225613.

## Introduction

Anterior cruciate ligament (ACL) injury is one of the main injuries in sporting activities with an incidence of nearly 32,000 victims per year in France (1), especially in young patients or athletes. This injury is rather unusual in the general population and can occur during most physical and sporting activities, particularly in activities which involve rotation constraints of the lower limbs such as football, rugby, handball, basketball, volleyball, and especially skiing (2). The ACL does not recover spontaneously due to its poor vascularity, and rupture of this ligament may result in short-term, medium-term, and long-term complications and poor functional prognosis. Instability of the knee and subsequent postural control impairments can occur in sporting activities, especially pivot sports, or during activities of daily living (3). This instability can affect other structures of the knee by damaging the meniscus which acts as a shock absorber. These events can also damage articular cartilage of the femur and the tibia leading to long-term osteoarthritis (4–7). Reconstruction of ACL by auto-graft tendon is the optimal treatment for these injuries, offering maximal joint stability with minimal surgical risk (3). Several studies have shown recovery of postural stability (8–10), which can be explained by the regeneration of sensory neurons after reconstruction of ACL (11). Among the reconstruction techniques, a hamstrings autograft uses the tendons of the gracilis and semitendinosus muscles. Once removed, these tendons are folded in half in order to obtain a new ligament with four strands. Among other techniques, a tape locking screw (TLS), developed by Collette in Brussels in 2001 (12), is nowadays used in France in 10% of cases (2). This technique uses only one fragment of the tendon of the semitendinosus muscle (the tendon of the gracilis may nevertheless sometimes be used). The graft is placed in small diameter bone tunnels with suspension strips and an interference screw. Finally, the Kenneth Jones (KJ) technique relies on the removal of the central third of the patellar tendon with a bone rod at each end, at the top of the femur and at the bottom on the tibia (13). Regardless of the technique, the graft taken is calibrated and then prepared before being inserted and fixed in both tibial and femoral bone tunnels. Surgery has to be followed by a long process of neuromotor reprograming through rehabilitation. Several rehabilitation techniques are used and studies have been carried out to compare the effects of rehabilitation in water versus more traditional rehabilitation on land. Tovin et al. (14) have shown that 2 months after surgery, aquatic exercises may not be as effective than on land for muscle strength but that water has a beneficial effect on edema and pain decrease. Zamarioli et al. (15) showed that during 9 weeks of rehabilitation, patients undergoing aquatic rehabilitation tended to recover faster than those who had undergone conventional land rehabilitation on clinical parameters such as pain, range of motion, muscle strength, swelling, and muscle mass circumference. Tovin et al. (14) suggested that future studies should analyze the effectiveness of a program that combines traditional and water-based exercises over a longer follow-up period.

The main objective of this study was to compare an innovative rehabilitation protocol with an “aquatic part” (balneotherapy) and a “dry part” and a conventional rehabilitation protocol (1) in terms of dynamics of recovery and development of the proprioceptive skills in athletes with ACL reconstruction. A secondary objective was to compare both groups in terms of functional improvement, i.e., pain, joint amplitude, muscular strength, and walking performance.

## Patients and Methods

### Setting and Participants

Sixty-seven patients aged from 18 to 49 years participated in this study. The volunteers underwent surgery by orthopedic surgeons from the University Hospital of Nancy and private clinics around Nancy, East of France. Among the 67 patients, none have undergone surgical complications.

All subjects were leisure or competitive sportsmen (amateur or professional). All of them had chronic knee instability and the indication of a first-line ACL reconstruction using TLS, semitendinosus or gracilis tendons or KJ was given. Exclusion criteria were history of neurological disease (stroke, degenerative diseases of the central nervous system or the peripheral nervous system), medication for psychotropic or antihypertensive purposes, contraindications to aquatic activities (especially cutaneous), and recent sprains less than 3 months in the lower limbs that could interfere with postural control.

This study is multicenter, prospective, randomized, and controlled with a group of patients following conventional rehabilitation (level of evidence I). All participants gave written informed consent before the randomization. The study was approved by the French Medical Ethical Committee (Comité de Protection des Personnes de Lorraine), realized in structures (Nancy-Thermal) agreed for research (Agence Régionale de Santé), and registered in ClinicalTrials.gov with the identifier: NCT02225613.

### Randomization and Interventions

**Figure 1:**
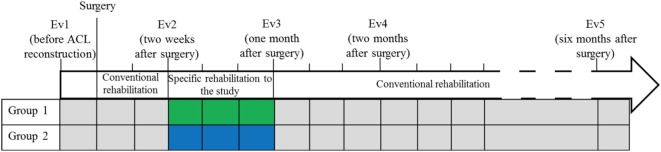
Timeline of the rehabilitation protocol. Phases of conventional rehabilitation and specific rehabilitation to the study. Group 1: conventional rehabilitation group; Group 2: innovative rehabilitation protocol with a conventional part and an aquatic part.

All patients began the same conventional rehabilitation protocol according to the recommendations of the National French Health Authority (HAS), until tissues healing (3). For 3 weeks, starting from the 15th postoperative day, subjects were randomized into two rehabilitation groups. To implement randomization, a statistician assigned numbers in two groups, each number corresponding to a patient. Sealed envelopes with the numbers were then prepared by the study Promotor (Direction for Research of the University Hospital who is not investigator of the study). No patient refused the group into which they were assigned. The first group (Gr1, *n* = 35; mean age = 29.91 ± 7.70 years; 21 males) followed a conventional rehabilitation protocol (according to HAS recommendations) and the second group (Gr2; *n* = 32; mean age = 28.22 ± 7.38; 26 males) followed an innovative rehabilitation protocol with a reduced conventional part and an aquatic part. The total duration of patient care was the same between the two groups regardless of the rehabilitation protocol followed: 45 min, once a day, five times a week, from Monday to Friday, for 3 weeks. After these 3 weeks, all patients returned to a conventional rehabilitation (according to HAS recommendations) (3) (Figure 1).

### Outcomes and Follow-up

#### Postural Control Statement

**Figure 2:**
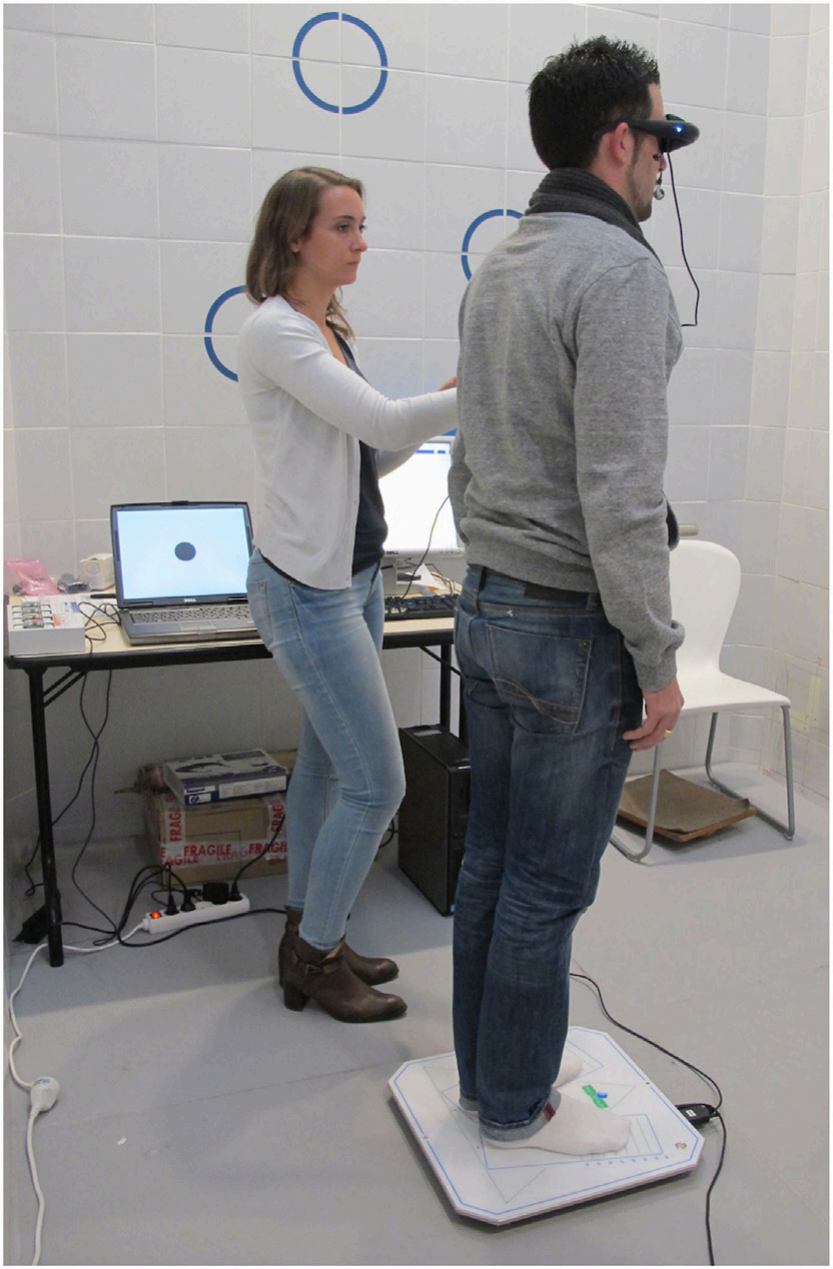
Postural control analysis on a posturography platform (Medicapteurs, Balma, France). Virtual reality goggles (RM Ingénierie, Rodez, France).

**Figure 3:**
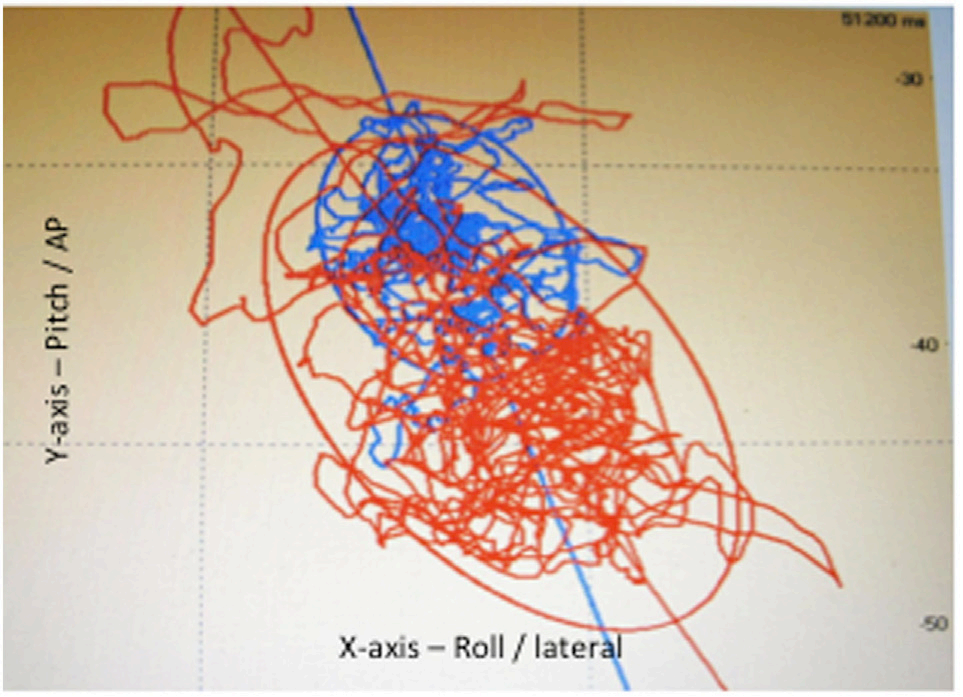
Posturography: statokinesigram, sway path traveled and area covered (confidence ellipse covering 90% of the points) by the center of foot pressure, in eyes open (blue) and eyes closed (red) conditions.

Postural control tests, main endpoint, were carried out in a specially designed sound proof room devoted to posturography recordings. A vertical force platform, fitted with three strain-gauge force transducers (Medicapteurs, Balma, France) was used to perform posturography and to provide a measurement of the body sway in terms of displacement of the center of foot pressure (CoP) in a two-dimensional horizontal plane (recording time: 25.6 s, acquisition frequency: 40 Hz) (Figure 2). The signals from the transducers were amplified, converted from analog into digital form and then recorded on a computer. The sway path traveled and area covered by the CoP trajectory were used to quantify postural sway (Figure 3). The mean shift on the X-axis was also used to quantify the lateralization and its side. Each subject was asked to stand upright on the platform, barefoot, feet abducted at 30°, heels separated by 3 cm, arms along the body, remaining as stable as possible and breathing normally in six conditions (16–18) to test somatosensory cues (19) (Table 1).

**Table 1:** Postural control test.

Postural control test		

Name	Situation	Sensory consequences
**Conditions**		
Condition 1 (C1)	Eyes open on firm support	–
Condition 2 (C2)	Eyes closed on firm support	Vision absent
Condition 3 (C3)	Vision altered (virtual reality goggles), on firm support	Altered vision
Condition 4 (C4)	Eyes open on foam support	Altered proprioception
Condition 5 (C5)	Eyes closed on foam support	Vision absent, altered proprioception
Condition 6 (C6)	Vision altered (virtual reality goggles) on foam support	Altered vision and proprioception

**Name**	**Pair**	**Significance**

***Ratios***		
Somatosensory (R_SOM_)	C2/C1	Question: does sway increase when visual cues are removed?
		Lower scores: better use of somatosensory references
Visual (R_VIS_)	C4/C1	Question: does sway increase when somatosensory cues are inaccurate?
		Lower scores: better use of visual references
Vestibular (R_VEST_)	C5/C1	Question: does sway increase when visual cues are removed and somatosensory cues are inaccurate?
		Lower scores: better use of vestibular cues

*Determination of the six conditions and significance of sensory ratios*.

In order to evaluate participants’ ability to adapt and adjust balance control performance correctly and rapidly to change of external and internal constraints, a mean equilibrium score (MES) was calculated by adding the scores of each condition, and dividing that sum by six [MES = (sum C1–C6)/6], low values being representative of good postural control (20–22). Each score was adjusted to score area in C1 to identify the significance of each sensory system influencing postural control, ratio C2/C1 representing the somatosensory contribution to postural control (R_SOM_), ratio C4/C1, the visual contribution (R_VIS_) and ratio C5/C1, the vestibular contribution (R_VEST_) (23, 24) (Table 1). A lower ratio of a sensory input is representative of a better use of this sensory input.

All patients were submitted to postural control tests before surgery (Ev1), and four times after the beginning of rehabilitation; at 2 weeks (Ev2) (corresponding to the beginning of the specific rehabilitation to the study), at 1 month (Ev3) (corresponding to the end of the specific rehabilitation to the study), at 2 months (Ev4) and at 6 months (Ev5) (Figure 3).

#### Clinical Parameters Statement

The functional improvement, secondary endpoints, was evaluated by different clinical assessments:

(1) The intensity of affected knee joint pain was assessed with the Visual Analog Scale (VAS) for pain. The VAS consisted of a 100 mm line whose endpoints were designated as “no pain” (at 0 mm) and “unbearable pain” (at 100 mm), respectively. Patients were requested to locate the level of knee pain on the line with a small vertical mark (25, 26).
(2) The proprioception test was carried out in a sitting position, on an examination table, without contact with the base of the lower half of the posterior part of the thigh. The tested member was placed on a skateboard. Flexion and extension movements were realized with the patients’ eyes closed. The lower limb was passively flexed or extended by the physiotherapist at an arbitrary angle before being returned passively to the neutral position. Patients were instructed to flex actively the tested limb to the same angle than the passive position determined by the physiotherapist (27–31). The error was measured in degrees by an inclinometer placed on the proximal part of the tibia below the anterior tuberosity.
(3) Joint amplitudes were evaluated in flexion and extension, active and passive (28, 32, 33).
(4) Trophicity refers to all the mechanisms and processes involved in the nutrition of organs and tissues. This test consisted of digital pressure on the edema after which it was observed whether the imprint left by the finger disappeared rapidly or not. This test was positive if the impression disappeared slowly (28).
(5) Muscle strength testing was performed in knee extension to evaluate the strength of the femoral quadriceps, and then in knee flexion to assess strength of the hamstrings. A score ranging from 0 (no contraction) to 5 (normal contraction) was attributed to muscle strength (28). An isokinetic test allowed evaluating parameters such as strength, muscle deficit, and hamstring/quadriceps ratio. The evaluation was performed on the contralateral and ipsilateral limbs in order to establish a comparison of the muscle strength of the two limbs at speeds of 60°s^−1^ and then 180°s^−1^.
(6) The 6 min walk test consisted of measuring the greatest possible distance that a patient could walk at their own pace in 6 min (34).
(7) Patients had to respond to the Lysholm-Tegner (35), International Knee Documentation Committee (IKDC) (36), and Knee injury and Osteoarthritis Outcome Score (KOOS) (37) questionnaires.

For each of the five evaluation times, the clinical tests performed are presented in Table 2.

**Table 2:** Tests schedule.

	Ev1	Ev2	Ev3	Ev4	Ev5
Posturography	x	x	x	x	x
Visual Analog Scale (VAS) pain	x	x	x	x	x
Proprioception test	x			x	
Joint amplitudes		x	x	x	x
Trophicity		x	x	x	x
Muscular strenght		x	x	x	x
Isokinetic test					x
6 min walk test			x		x
Questionnaires (Lysholm-Tegner, International Knee Documentation Committee, Knee injury and Osteoarthritis Outcome Score)	x				x

*Ev1: evaluation before anterior cruciate ligament reconstruction; Ev2: 2 weeks after surgery; Ev3: 1 month; Ev4: 2 months; Ev5: 6 months*.

### Statistical Analysis

Qualitative data were expressed in terms of number (*n*) and percentage (%). Quantitative data were expressed as mean and SD.

A cross-sectional analysis was used to compare the two rehabilitation groups at each evaluation time. Frequency comparisons in independent series were performed with the Chi squared test. Comparisons of means between the two rehabilitation groups were done using the Student’s *t* test for independent series. The ANOVA variance analysis was used to study the relationships between a quantitative dependent variable and several independent explanatory variables. Due to the small sample size tested on the isokinetic test, a nonparametric Mann–Whitney test was used to make comparisons between the two groups. A probability level *p* ≤ 0.05 was considered significant.

A longitudinal analysis was used to assess the progression of patients in a given group between two evaluations. Comparisons of mean values between the different evaluations in the same group were made by the Student’s *t* test for paired series. The ANOVA variance analysis was used to study the relationships between the difference between two evaluations and an independent explanatory variable. The McNemar analysis was used with ordinal variable to compare frequencies across time in each group. A probability level *p* ≤ 0.05 was considered significant except for the results of the posturography test. To take into account multiple comparisons, the Bonferroni procedure was applied to pairwise comparisons with a significant *p* < 0.05/5 = 0.01 adjustment due to the five evaluations.

The SPSS Statistics software (IBM, Armonk, NY, USA) version 23.0 was used for all analyzes.

## Results

### Participants

No statistically significant difference was observed for anthropometric variables (age, sex, height, weight, body max index) between the two groups of patients at baseline (Table 3). No significant difference was observed between the two rehabilitation groups regarding the surgical techniques used (*p* = 0.427).

**Table 3:** Baseline anthropometric characteristics of patients with conventional rehabilitation (Group 1) protocol and innovative rehabilitation protocol (Group 2).

	Group 1 (*n* = 35)	Group 2 (*n* = 32)	*p-*value
	Mean ± SD	Mean ± SD	Student’s *t* test
Age (years)	29.91 ± 7.70	28.22 ± 7.38	0.364
Height (m)	1.72 ± 0.08	1.76 ± 0.10	0.064
Weight (kg)	69.59 ± 11.55	73.34 ± 13.54	0.225
BMI (kg.m^-2^)	23.46 ± 2.70	23.44 ± 2.57	0.971

	***n* (%)**	***n* (%)**	**Chi2**

Sex, males	21 (60.00)	26 (80.25)	0.058

*SD, standard deviation; BMI, body mass index*.

### Postural Control

No statistically significant differences of the MES of the sway path traveled and the area covered by the center of foot pressure were observed between the two rehabilitation groups at the five evaluations. No statistically significant differences of R_VIS_ and R_VEST_ were observed between the two rehabilitation groups at the five evaluations whereas R_SOM_ score was significantly lower in Gr2 than in Gr1 (*p* = 0.008) at Ev5, reflecting a greater use of somesthesia in balance control (Table 4). Patients of the two groups rely on the contralateral limb to the surgery to the evaluation at Ev1 (*p* = 0.002), Ev2 (*p* < 0.001) and Ev3 (*p* = 0.001). Gr1 rely more than Gr2 on the contralateral limb to the surgery at Ev1 (*p* = 0.037) and Ev3 (*p* = 0.039).

**Table 4:** Comparison of the postural control between the two groups of rehabilitation.

	Group 1 (*n* = 35)	Group 2 (*n* = 32)	Student’s *t* test
	
	Mean ± SD	Mean ± SD	*p-*value
**Evaluation 1**			
Area (mm^2^)	633.78 ± 229.36	767.64 ± 459.74	0.131
Sway path (mm)	660.53 ± 215.01	649.83 ± 235.13	0.846
R_SOM_	1.46 ± 0.80	1.60 ± 0.98	0.506
R_VIS_	3.69 ± 2.42	3.81 ± 1.59	0.812
R_VEST_	12.11 ± 5.73	16.09 ± 11.36	0.081
**Evaluation 2**			
Area (mm^2^)	696.13 ± 251.07	740.90 ± 348.25	0.546
Sway path (mm)	729.58 ± 187.42	732.20 ± 231.81	0.959
R_SOM_	1.35 ± 0.54	1.63 ± 1.06	0.176
R_VIS_	2.44 ± 1.32	2.51 ± 1.26	0.825
R_VEST_	7.88 ± 5.08	8.22 ± 4.95	0.784
**Evaluation 3**			
Area (mm^2^)	610.53 ± 205.35	656.21 ± 213.39	0.375
Sway path (mm)	676.18 ± 183.81	631.80 ± 187.17	0.331
R_SOM_	1.46 ± 1.02	1.54 ± 1.15	0.746
R_VIS_	2.83 ± 1.86	2.59 ± 1.42	0.548
R_VEST_	8.17 ± 3.81	7.52 ± 3.51	0.478
**Evaluation 4**			
Area (mm^2^)	556.13 ± 173.78	595.04 ± 205.83	0.243
Sway path (mm)	612.64 ± 165.88	589.98 ± 176.82	0.590
R_SOM_	1.45 ± 0.67	1.31 ± 0.61	0.356
R_VIS_	3.27 ± 1.63	2.95 ± 1.44	0.404
R_VEST_	8.07 ± 3.88	7.92 ± 4.14	0.880
**Evaluation 5**			
Area (mm^2^)	583.72 ± 196.31	639.65 ± 286.87	0.352
Sway path (mm)	629.10 ± 220.00	603.67 ± 198.25	0.622
R_SOM_	1.62 ± 0.87	1.14 ± 0.50	**0.008**
R_VIS_	4.32 ± 3.25	3.09 ± 2.27	0.080
R_VEST_	11.51 ± 7.26	9.57 ± 7.86	0.297

*SD, standard deviation*.

Significant differences were showed between Ev1 and Ev2 for R_VIS_ and R_VEST_ in both groups. Patients used more visual and the vestibular input at Ev2 to maintain their balance control (Gr1: R_VIS_, *p* = 0.004; R_VEST_, *p* < 0.001; Gr2: R_VIS_, *p* = 0.001; R_VEST_, *p* < 0.001). In Gr1, the area decreased between Ev2 and Ev3 (*p* = 0.004) and between Ev3 and Ev4 (*p* = 0.001), and the sway path decreased between Ev3 and Ev4 (*p* = 0.001). In Gr2, the sway path increased between Ev1 and Ev2 (*p* = 0.003) and decreased between Ev2 and Ev3 (*p* = 0.001). Gr1 relied more on the limb contralateral to the operated limb between Ev1 and Ev2 (*p* < 0.001), whereas they relied less on the contralateral limb between Ev2 and Ev3 (*p* < 0.001) and Ev1 and Ev5 (*p* = 0.010). Gr2 relied more on the limb contralateral to the surgery between Ev1 and Ev2 (*p* < 0.001), whereas they relied less on the contralateral limb between Ev2 and Ev3 (*p* < 0.001).

### Clinical Parameters

(1) No statistically significant difference in pain was observed between the two rehabilitation groups over time. In Gr1, pain decreased between Ev2 and Ev3 (*p* = 0.007) and Ev1 and Ev5 (*p* = 0.039). In Gr2, pain increased between Ev1 and Ev2 (*p* = 0.027), then decreased between Ev2 and Ev3 (*p* < 0.001), Ev4 and Ev5 (*p* = 0.030), and Ev1 and Ev5 (*p* = 0.012).
(2) No statistically significant difference in proprioception was observed between the two groups over time. In Gr2, an improvement of proprioception in flexion was observed at Ev4 (*p* = 0.002).
(3) No statistically significant difference in joint amplitude was observed between the two groups over time. In the two groups, an improvement in active and passive flexion was observed between Ev2 and Ev3 (*p* < 0.001), Ev3 and Ev4 (*p* < 0.001), Ev4 and Ev5 (*p* < 0.001). In Gr1, an improvement in passive extension was observed between Ev2 and Ev3 (*p* = 0.015) and Ev3 and Ev4 (*p* = 0.006), and an improvement in active extension was observed between Ev4 and Ev5 (*p* < 0.001). In Gr2, an improvement in active extension was observed between Ev2 and Ev3 (*p* = 0.008), Ev3 and Ev4 (*p* = 0.038), and Ev4 and Ev5 (*p* = 0.002). An improvement in passive extension was observed between Ev2 and Ev3 (*p* = 0.021) and Ev4 and Ev5 (*p* = 0.006) in Gr2.
(4) No statistically significant difference in trophicity was observed between the two groups over time. An improvement in trophicity was observed in the two groups between Ev2 and Ev3 (Gr1: *p* = 0.039; Gr2: *p* = 0.006) and Ev4 and Ev5 (Gr1: *p* = 0.004; Gr2: *p* = 0.035).
(5) Statistically significant differences in quadriceps muscle strength were observed at Ev4 (*p* = 0.015) and Ev5 (*p* = 0.027) between the two groups. At Ev4 in Gr2, 14 patients obtained a score of 5 (43.75%), 17 a score of 4 (53.13%) and 1 a score of 3 (3.12%) versus 5 a score of 5 (14.29%), 25 a score of 4 (71.43%) and 1 a score of 3 (2.85%) in Gr1. At Ev5 in Gr2, 24 patients obtained a score of 5 (75%) and 8 a score of 4 (25%) versus 17 a score of 5 (48.57%) and 18 a score of 4 (51.43%) in Gr1. Statistically significant differences in internal hamstring muscle strength were observed at Ev2 (*p* = 0.026). In Gr2, 13 patients obtained a score of 4 (40.63%), 14 a score of 3 (43.75%), and 5 a score between 0 and 2 (15.62%) versus 8 a score of 4 (22.86%), 26 a score of 3 (74.29%) and 1 a score between 0 and 2 (2.85%) in Gr1. Statistically significant differences in external hamstring muscle strength were observed at Ev5 (*p* = 0.019) between the two groups. In Gr2, 22 patients obtained a score of 5 (68.75%), 9 a score of 4 (28.13%) and 1 a score of 3 (3.12%) versus 12 a score of 5 (34.29%), 21 a score of 4 (60.00%) and 2 a score of 3 (5.71%) in Gr1. The statistical analysis showed an increase in the muscle strength of the quadriceps between Ev2 and Ev3 (*p* = 0.006) and Ev3 and Ev4 (*p* = 0.039) in Gr1. The muscle strength of the hamstring increased between Ev2 and Ev3 (*p* = 0.001) and Ev4 and Ev5 (*p* = 0.031). In Gr2, the muscle strength of the quadriceps increased between Ev2 and Ev3 (*p* < 0.001), and the muscle strength of the hamstring increased between Ev2 and Ev3 (*p* < 0.001). At Ev5, the muscle strength of the quadriceps developed in isokinetic testing was significantly greater in Gr2 at either 60°s^−1^ (*p* = 0.027) or 180°s^−1^ (*p* = 0.047). Moreover, muscle deficit of the quadriceps of the ipsilateral limb compared to the contralateral limb was lower in Gr2 (*p* = 0.020).
(6) A statistically significant difference in the 6 min walk test was observed between the two groups at Ev3 (*p* = 0.025). Gr1 traveled 425.27 ± 158.45 m against 506.59 ± 127.70 m for the Gr2. 6 min walking performance significantly increased at Ev5 compared to Ev3 in the two groups (*p* < 0.001) and there was no significant difference at Ev5.
(7) No statistically significant difference for questionnaires was observed between the two groups. An improvement in the functional Lysholm-Tegner score was observed between Ev1 and Ev5 in Gr1 (*p* < 0.001) and Gr2 (*p* = 0.004). Statistical analysis showed an improvement in the IKDC functional score in both groups (*p* < 0.001). Significant improvement in knee function after injury was observed in both groups (*p* < 0.001). In both groups an improvement was observed for pain (Gr1, *p* = 0.001; Gr2, *p* = 0.016), daily life (Gr1, *p* < 0.001; Gr2, *p* < 0.001), quality of life (Gr1, *p* < 0.001; Gr2, *p* < 0.001) and an increase in sports and leisure activities (Gr1, *p* < 0.001; Gr2, *p* < 0.001).

## Discussion

This study aimed to compare an innovative rehabilitation protocol combining a dry and an aquatic part with a conventional rehabilitation protocol with the same total duration of patient care, in terms of dynamics of recovery, development of proprioceptive skills and functional improvement in athletes with ACL reconstruction. The main results of this study showed that for the same quality of postural control, patients who followed the innovative rehabilitation protocol had less lateralization at the side contralateral to the surgery than patients who followed the conventional rehabilitation protocol after the specific period of rehabilitation. The innovative group also relied more on somesthesia 6 months after surgery. Secondary results showed that, although muscle strength increased in both rehabilitation groups during follow-up, patients who followed the innovative rehabilitation protocol had greater muscle strength of the external hamstring 6 months after surgery and greater muscle strength of the quadriceps 2 months and 6 months after surgery. This difference in muscle strength of the quadriceps 6 months after surgery was confirmed by the isokinetic test. This test confirmed a difference in muscle strength between the two groups at the rate of 60°s^−1^ as well as at the rate of 180°s^−1^ and a lower deficit of the quadriceps in the innovative rehabilitation protocol group. On the other hand, although walking distance in 6 min increased in both groups between the 1st month and the 6th months after surgery, patients who followed the innovative rehabilitation protocol walked a greater distance 1 month after surgery. Finally, although both rehabilitation groups had similar proprioceptive performances before and 2 months after surgery, patients who followed the innovative rehabilitation protocol showed an improvement of proprioception at the ipsilateral limb 2 months after surgery compared to before surgery.

The main endpoint showed that for the same quality of postural control 6 months after surgery, patients who followed the innovative rehabilitation protocol increased the weight of somesthesia more than the conventional rehabilitation protocol group. The conventional group did not increase somesthesia in the course of evaluations. This means that patients who followed innovative rehabilitation had developed more proprioceptive capacities during rehabilitation. On the other hand, in agreement with the study of Dauty et al. (38), 15 days after surgery, patients of both groups relied more heavily on the contralateral limb as demonstrated by the posturography test. This compensation necessity decreased in both groups one month after surgery and was less needed in patients receiving the aquatic rehabilitation protocol than in patients who had followed the conventional rehabilitation protocol. Reduced gravity and water buoyancy decrease the detrimental effects of weight bearing and impact forces on joint structure (39) and the Archimedes’ principle allows an early and progressive loading by lowering the immersion level (40). This progressive loading can explain the reduced lateralization in the patients who followed the protocol of innovative rehabilitation one month after surgery.

In this study, measurements of joint amplitudes, trophicity, pain assessment, and Lysholm-Tegner, IKDC and KOOS questionnaires did not show any significant difference between the two groups. The evolution of these parameters during the 6 months of follow-up showed similarities in the two groups. First, an improvement in the flexion and extension of the knee joint was observed in the two rehabilitation groups between the 15th day and the 1st postoperative month, between the 1st month and the 2nd postoperative month, and between the 2nd month and the 6th postoperative month. In rehabilitation, the gain in joint amplitude is obtained initially by soft techniques of recovery of the mobility. Then, massages of all the planes of articular sliding with kneading, frictions, and deep transverse massages make possible to avoid retractions. Passive and active mobilization allows for an increase in flexion by the use of supra-patellar supports, contraction-relaxation and the search for lateral sliding by manual support or contraction of the hamstrings.

We also compared pain reduction in the two groups. A reduction in pain was observed between the 15th day and the 1st postoperative month, and between the 2nd month and the 6th postoperative month in the group following the innovative rehabilitation protocol. The analgesic means used in rehabilitation such as circulatory massages, cryotherapy, or scar massages to avoid the formation of tissue adhesions, all participate to this reduction of pain. This is supported by the fact that pain 6 months after surgery is lower than the pain experienced before surgery.

An improvement in trophicity was found between the 15th day and the first postoperative month, as well as between the 2nd month and the 6th postoperative month in both groups. Massages allowed lymphatic drainage and decreased edema. Finally, the Lysholm-Tegner, IKDC, and KOOS questionnaires showed an increase in scores between preoperative evaluation and evaluation 6 months after surgery in both rehabilitation groups (41). Thus, patients judged the functionality of their knee to be better 6 months after surgery than before surgery. They also have less pain, improve their daily life and quality of life, and take up recreational activities and sports activities.

Patients who followed the innovative rehabilitation protocol had significantly greater muscular strength than patients who followed the conventional rehabilitation protocol 2 months and 6 months after surgery. Several properties of the aquatic environment can explain this difference. First, a body immersed in water is subjected to a resistance effect called drag force and turbulence (40). This resistance to advancement increases with the square of the speed: the faster the body or part of the body moves, the greater the driving strength (42). In an aquatic environment, when a patient feels pain, he can stop his movement; the force then falls precipitously because the viscosity of the water dampens the movement almost instantaneously. This allows better control of muscle building (40). Second, after any intra-articular injury of the knee, resting of the quadriceps is observed. The Archimedes’ principle allows the quadriceps to be stressed in discharge, and therefore, a soft muscle building. Third, lowering the body’s apparent weight allows early loading, and therefore, progressive muscular strengthening by lowering the immersion level (40, 42). In addition, water temperature causes a muscular heating which can favor the mobilization of the operated member and thus allow indirectly a muscular building. Hydrostatic pressure also plays an indirect role in muscle strengthening by promoting venous and lymphatic return. As a result of this, contractures (i.e., the defense mechanism that takes place after a trauma to block the traumatized zone) will disappear and thus allow a progressive mobilization, and therefore, a muscular effort of the initially contracted area. However, it was reported that electromyographic activity of under water exercises was decreased as compared to similar exercises performed on dry land (44–46). Moreover, in 1994, Tovin et al. (14) had already compared rehabilitation exercises in the aquatic environment with dry rehabilitation exercises in patients having surgery of the ACL injury and showed that aquatic exercises were not as effective as dry exercises for strength gain and muscle trophicity. Nevertheless, the studies on the subject are contradictory since Zamarioli et al. (15) showed that patients following an aquatic rehabilitation had a better recovery of the muscular strength. Our study had the specificity of combining dry aquatic rehabilitation and aquatic rehabilitation. Dry exercises and exercises in the aquatic environment differ in nature and this could explain this difference in muscular strength in favor of patients who have followed the protocol of innovative rehabilitation. The Archimedes’ principle allows standing exercises with a normal motor pattern for the patient, which is directly transferable on the land and which allows a better dry muscle strengthening than exercises that would be made in seated or lying down positions. Despite this difference in muscle strength between the two rehabilitation groups, a progressive improvement was observed in both groups during the 6 months of follow-up. Our study is in agreement with other studies that have shown an improvement in muscle strength and a reduction in the difference in muscle strength between the operated limb and the contralateral limb after different rehabilitation programs (41, 47–49). This progressive improvement can be explained by a muscular building process. On the one hand, muscular rehabilitation aims at combating muscular amyotrophy. On the other hand, the hamstrings play the role of braking the last degrees of extension and also stabilizing the joint when the knee is bent under load. At the end of rehabilitation, muscular building favors exercises derived from sporting activities.

This study showed that patients who followed the innovative rehabilitation protocol walked a greater distance in 6 min than patients who followed the conventional rehabilitation protocol 1 month after surgery. With water immersion, gravitational forces can be partially or completely compensated so that only the forces of the couple act on the injured site. For example, for an immersion up to the shoulders, the body’s apparent weight is 15–20 kg and only a few active motor units are needed to make the movement possible. Due to the apparent decrease in body weight, progressive loading is made possible by lowering the immersion level (40, 43). Immersion thus allows active movements, which are automatically transferred to walking pattern in land conditions. In addition, 6 months after surgery, there was no significant difference between the two rehabilitation groups, suggesting that patients who followed the innovative rehabilitation protocol recovered capacities faster than the group following the conventional rehabilitation protocol but patients who followed conventional protocol did catch up by 6 months.

The proprioception test revealed no significant difference between the two rehabilitation groups before surgery and 2 months after surgery. Nevertheless, after comparing the results obtained before and 2 months after surgery, this study showed that patients who followed the innovative rehabilitation protocol significantly reduced the proprioceptive deficit in flexion of the operated knee after surgery. The aquatic environment forces the patient to maintain balance under new conditions. These conditions allow the stimulation of the proprioceptive pathway by creating situations of imbalance that the patient must gradually control. For example, a patient standing with a swimming board placed under the foot develops proprioceptive information. As the swimming board tends to rise to the surface, the patient has to demonstrate good coordination to maintain it, strengthening his neuromuscular vigilance and work in balance (40). Moreover, hydrostatic pressure associated with the environment viscosity is the source of external sensory stimuli. Immersing part of the body results in developing a better perception of the position of the limbs. Similarly, the resistance to displacement created swirling sensations, which enhances exteroceptive or even proprioceptive information, and thus, allows a better awareness of the body pattern (40).

This work has several limitations. This study required 14 physiotherapists but to avoid variations by ensuring repeatability, harmonization of practices was carried out before the first patient was enrolled. Another limitation of this study was due to auto-questionnaires. This always results in subjectivity of responses and it is possible that some patients may overestimate or underestimate the functional capacity of the knee. The duration of management in aquatic rehabilitation specific to the study was short. Nevertheless, this duration had been chosen to align with the model of spa treatment in France, which lasts an average of 3 weeks.

## Conclusion

This study shows that the innovative rehabilitation protocol (therapy incorporating both a dry and aquatic segment) improves proprioception and limits overcompensation on the limb contralateral to the operated limb. Even if patients undergoing a conventional rehabilitation protocol recover the delay after 6 months of surgery, faster and better recovery of knee functionality, following aquatic rehabilitation would in the short-term prevent injury to the contralateral limb as a result of overcompensation, and serve in the longer term to reduce the risk of osteoarthritis. The effectiveness of such rehabilitation could also enable patients to recover social, physical and professional activities earlier, which would also be of economic benefit, in particular with a reduction in work absence.

## Ethics Statement

All participants gave written informed consent before the study, which was approved by the French Medical Ethical Committee (Comité de Protection des Personnes de Lorraine) and registered in ClinicalTrials.gov with the identifier: NCT02225613.

## Author Contributions

All authors listed have made substantial, direct, and intellectual contribution to the work and approved it for publication.

## Conflict of Interest Statement

The authors declare that the research was conducted in the absence of any commercial or financial relationships that could be construed as a potential conflict of interest.
